# Immature Teratoma With Metastatic Gliosis

**DOI:** 10.7759/cureus.22748

**Published:** 2022-03-01

**Authors:** Ange Ahoussougbemey Mele, Shivang Danak, Ezra Ellis, Andrew Green

**Affiliations:** 1 Internal Medicine, Northeast Georgia Medical Center Gainesville, Gainesville, USA; 2 Pathology, Northeast Georgia Medical Center Gainesville, Gainesville, USA; 3 Obstetrics and Gynecology, Northeast Georgia Medical Center Gainesville, Gainesville, USA

**Keywords:** tumor staging, cancer immunotherapy, chemotherapy agents, metastatic ovarian cancer, immature teratoma

## Abstract

Immature teratomas are rare malignant tumors of the ovary. They are made of immature components of germ cell origin. The incidence of immature teratomas is highest in young adults aged 18 to 39. The prognosis heavily depends on the International Federation of Gynecology and Obstetrics (FIGO)* *staging system and is influenced by factors such as cell type, tumor grade, capsular rupture, and metastatic risk factors. Initial treatment is complete surgical resection. When indicated, platinum-based adjuvant chemotherapy with bleomycin, etoposide, and cisplatin (BEP) is the treatment of choice. Next-generation sequencing of the tumor can influence treatment in the recurrent setting. Temozolomide is an alkylating agent used to target high-grade gliomas. Bevacizumab is a targeted therapy that interferes with the process of angiogenesis by inhibiting vascular endothelial growth factor (VEGF).

We report a 36-year-old female who presented with a 17.6cm x 10.5cm x 24.2cm intraabdominal mass and ascites. Upon tumor resection, she was found to have a stage IIIa, grade 2 immature teratoma of the left ovary, with glial tissue being the metastatic cell type. Disease progression continued despite treatment with BEP. She was then treated experimentally with six months of bevacizumab and temozolomide, given its rarity and targeted therapy for glial tissue. Despite monoclonal antibody therapy, the tumor progressed again and was treated with docetaxel and gemcitabine. A repeat CT of the chest, abdomen, and pelvis demonstrated scattered peritoneal implants that were increasing in size. Chromosome analysis was performed and revealed somatic mutations of MLH1, MSH2, MSH6, and PD-L1. The patient has requested a break from chemotherapy but will be treated with direct immunotherapy when she restarts.

This case’s importance lies in its rarity because fewer than 10 cases of immature teratomas with metastatic glial tissue are noted in the world’s literature. Furthermore, this is the first reported case of this cell type being treated with immunotherapy in the world literature.

## Introduction

Teratoma originates from the Greek word “teras,” which signifies monster. Immature teratomas are malignant tumors of the ovary and represent 1% to 3% of all germ cell tumors and 20% of malignant ovarian germ cell tumors [1]. In comparison, mature teratomas are benign. The grading system depends on the proportion of mature and immature neuroepithelial tissues, mitotic activity, and degree of differentiation. Per Iavazzo et al., grade 0 tumors contain solely mature tissue, whereas tumors of grades 1, 2, and 3 are mitotically active and possess limited, moderate, and large amounts of neuroepithelial tissue, respectively [2]. Following laparoscopic surgery, the pathological diagnosis of the excised tumor determines whether an ovarian tumor is mature or immature. Immature teratomas are composed of embryonic components, are 14 to 25 cm larger than mature cystic teratoma, and possess solid components in the cystic elements.

## Case presentation

We report a 36-year-old female with a medical history of Hashimoto’s thyroiditis on levothyroxine who presented with complaints of progressively worsening abdominal distention and clothes fitting tighter over the past two months. She also endorsed left lower quadrant abdominal and suprapubic pain. She reported her symptoms were associated with early satiety, constipation, nausea without emesis, and not being relieved by prescription probiotics. These symptoms prompted her to visit the emergency department. On physical examination, she had a distended abdomen with tenderness to palpation and a low-grade fever. Complete blood count was significant for anemia with a hemoglobin of 8.4 g/dL and hematocrit of 26.9%. Complete metabolic panel noted hypocalcemia of 7.5 mg/dL. Computed tomography (CT) of the abdomen and pelvis was significant for a bulky mass arising in the anterior pelvis and extending cephalad above the level of the umbilicus. The mass measured 17.6 cm transversely, 10.5 cm in the anteroposterior dimension, and 24.2 cm in the craniocaudal dimension. The lesion consisted of fluid and solid components with some calcifications and fat present. Furthermore, there was small to moderate free fluid in the posterior pelvis and lower abdomen bilaterally. 

The patient agreed to exploratory laparotomy and underwent left salpingo-oophorectomy, appendectomy, omentectomy, left pelvic and aortic lymph nodes sampling, and tumor debulking. Operative findings showed a large left-sided ovarian mass that was fleshy in nature. Frozen sections noted possible teratoma with stromal components concerning for malignancy; therefore, the patient was staged. Pathology results of specimens collected during the surgery showed grade 2 immature teratoma of the ovary, acute appendicitis, gliomatosis peritonei, peritoneal tumor and omentum, aortic and pelvic lymph nodes negative for metastatic tumor. The clinical stage showed the International Federation of Gynecology and Obstetrics (FIGO) stage IIIA1(i) calculated as stage IIIA2 (cT3a, cN0, cM0). 

**Figure 1:**
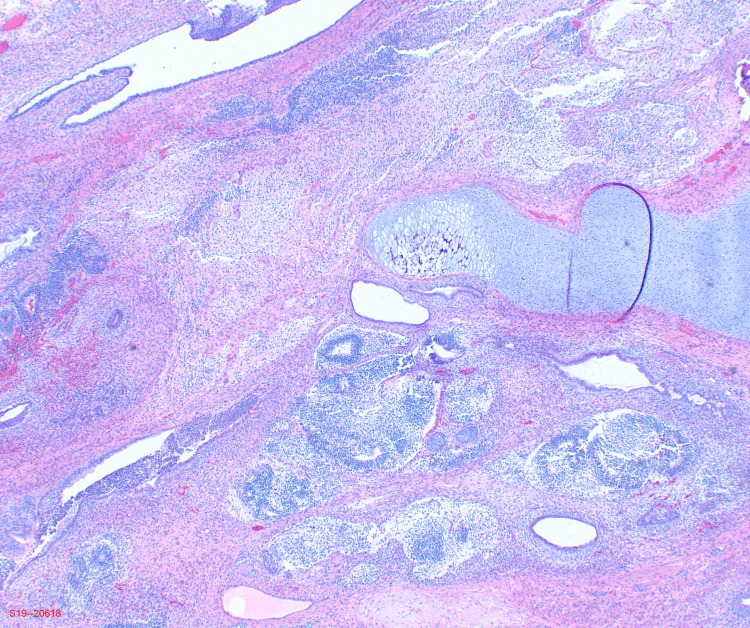
Histologic imaging showing cartilage, glandular and immature neural tissue.

**Figure 2:**
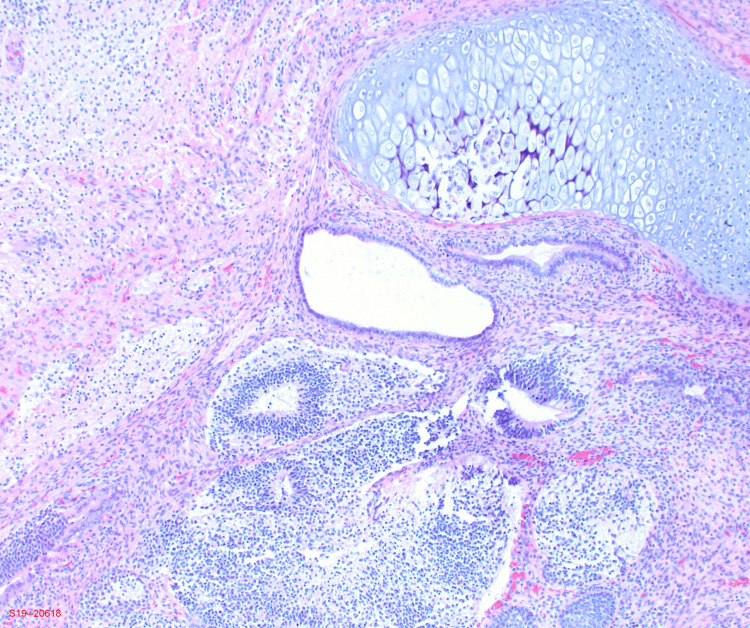
Histologic imaging showing cartilage, glandular and immature neural tissue.

The pathology results of the left ovary were notable for grade 2 immature teratoma of the ovary. The right pelvic peritoneum, omentum, abdominal fluid, and peritoneal tumor were remarkable for the presence of immature glial tissue. The left fallopian tube, appendix, left pelvic, and aortic lymph nodes were negative for tumor. Immunohistochemical stains were positive for glial fibrillary acidic protein, which is consistent with immature teratoma. In addition, mesothelial cells were positive for pankeratin, calretinin, and vimentin. As seen in Figures 1, 2, pathological imaging for the tumor was notable for cartilage, glandular and immature neural tissue. 

She was started on apixaban for deep venous thrombosis (DVT) prophylaxis and oxycodone as needed for pain. Additionally, she was started on bleomycin, etoposide, and cisplatin chemotherapy regimen every 21 days for four cycles. Repeat CT scan showed progression of disease with multiple soft tissue masses seen within the mesentery representing recurrence of the patient’s disease at three months post exploratory laparotomy and start of chemotherapy. It also noted ill-defined density within the pelvis posterior to the uterus, also indicating disease recurrence. To obtain a target, molecular testing was ordered. Subsequently, molecular testing and chromosome analysis were done to direct treatment. Approval was obtained, and the patient was started on temozolomide. 

Results showed that immunohistochemistry of the mass was positive for MLH1, MSH2, MSH6, and PD-L1 and variation of undetermined significance of BRCA1 and AXIN2. The patient was started on immunotherapy with bevacizumab through port-a-cath. At this point, she reported symptoms of shortness of breath accompanied by chest pain both at rest and with exertion, as well as bilateral pedal swelling. She also developed chemotherapy-induced hypertension. Following cardiology recommendations, the patient was started on carvedilol, amlodipine, and as-needed Lasix for bilateral pedal edema. An echocardiogram was also ordered as bevacizumab is known to cause hypertension and left ventricular dysfunction toxicity. The transthoracic echocardiogram showed mitral and tricuspid valve regurgitation. It also noted a normal left ventricular systolic function with an ejection fraction of 55%-60%. The patient’s current chemotherapy regimen, which included IV bevacizumab and temozolomide, was stopped, and she was started on gemcitabine and docetaxel. The patient was also informed of the palliative nature of the treatment. Three months later, the patient decided she would like to take a break from the chemotherapy, and the treatment was stopped. 

## Discussion

Immature teratomas are malignant tumors that occur in the first few decades of life. Tumor grading of immature teratomas is based on the amount of immature neural tissue present. The prognosis of immature teratomas depends mainly on the staging system provided by the International Federation of Gynecology and Obstetrics (FIGO) [3]. The other factors that influence prognosis are the tumor’s grade, its growth pattern, the occurrence of capsular rupture, and invasion of blood vessels. The yolk sac contained within the tumor has also been recognized as the source of alpha-fetoprotein and a predictor of the stage, grade, and rate of recurrence [4]. Javadi et al. reported the revised 2014 FIGO staging system for ovarian cancers [5]. This information has been summarized in Table 1. Our patient was found to have a FIGO stage IIIA1(i) calculated as stage IIIA2 (cT3a, cN0, cM0). This signifies she had microscopic extra pelvic peritoneal involvement. Javadi et al. further report that patients who present with an exclusively retroperitoneal lymph node involvement have a better prognosis than patients with abdominal peritoneal involvement, as is the case in this patient [5]. 

**Table 1:** Revised 2014 FIGO staging system Data collected from Javadi et al. [5] FIGO: the International Federation of Gynecology and Obstetrics

FIGO Staging System	Definition of Tumor Stage
Stage IA	A stage IA tumor is defined as one that is confined to one ovary or fallopian tube, has an intact capsule, and does not contain any tumor cells in ascites or washings.
Stage IB	A stage IB tumor involves both ovaries or fallopian tubes and is otherwise like a stage IA tumor.
Stage IC	A stage IC1 indicates an intraoperative spill; IC2 signifies the tumor ruptured before surgery or the tumor is on the ovarian or fallopian tube surface; IC3 indicates ascites and peritoneal washings were positive for the tumor.
Stage IIA	As such, a stage IIA tumor signifies that the tumor has extended to either the uterus, fallopian tubes, or a combination of both.
Stage IIB	A stage IIB signifies extension to other pelvic intraperitoneal tissues.
Stage IIC	The stage IIC has been eliminated in the 2014 revised FIGO staging system.
Stage IIIA1(i)	A stage IIIA1(i) indicates metastasis of less than or equal to 10 mm to the retroperitoneal lymph nodes.
Stage IIIA1(ii)	A stage IIIA1(ii) indicates metastasis of more than 10 mm to the retroperitoneal lymph nodes.
Stage IIIA2	A stage IIIA2 indicates microscopic extra pelvic peritoneal involvement.
Stage IIIB	A stage IIIB indicates macroscopic, extra-pelvic peritoneal metastasis of less than or equal to 2 cm with or without the involvement of retroperitoneal lymph nodes.
Stage IIIC	A stage IIIB indicates macroscopic, extra-pelvic peritoneal metastasis of more than 2 cm with or without the involvement of retroperitoneal lymph nodes.
Stage IV	Not Applicable
Stage IVA	Indicates pleural effusion with positive cytology
Stage IVB	A stage IVB indicates distant metastasis, including parenchymal metastasis to the liver, spleen, or extra-abdominal organs.

According to Iavazzo et al., fertility-sparing management is suggested as the standard of care of immature teratomas in young female patients [2]. Young female patients with immature teratomas should be educated about oncofertility and guided throughout the decision-making process. Per Iavazzo et al., the proposed algorithm for the management of immature teratoma is to first check the desire for the fertility of the patient. Once this step is completed, the grade of the tumor should be clarified, and radiologic imaging and tumor markers should be taken into consideration. If fertility is not desired, complete surgical resection may be performed. If fertility is desired, the next step in management depends on the grade of the tumor. For grade 1 tumors that have positive imaging and tumor markers, fertility-sparing surgery and comprehensive staging are completed. Grade 1 tumors with negative imaging and positive or negative tumor markers benefit from a conservative approach with observation, physical examination, and measurement of tumor markers every two to four months for the first two years, along with imaging as clinically indicated. For grade 2 to 3 tumors with positive imaging and tumor markers, fertility-sparing surgery with comprehensive staging or chemotherapy is the plan of care. For grade 2 to 3 tumors with negative imaging or positive or negative tumor markers, the patient should receive chemotherapy. If there is a response to treatment, the patient may be observed; if there is no response to treatment, tumor resection or observation should be considered. If there is further progression, platinum-based chemotherapy can be performed. In this case, our patient had a grade 2 moderately differentiated tumor with positive imaging and tumor markers. She agreed and underwent exploratory laparotomy with left salpingo-oophorectomy, appendectomy, omentectomy, left pelvic and aortic lymph nodes sampling, and tumor debulking. 

The patient was first started on bleomycin, etoposide, and cisplatin chemotherapeutic regimen. Etoposide inhibits DNA synthesis by interfering with the activity of topoisomerase II. It is predominantly active against cells in the late S and G-2 phases of the cell cycle. Cisplatin is an alkylating agent that acts in a cell-cycle nonspecific manner. Bleomycin is an antineoplastic antibiotic that binds to DNA and causes scissions of DNA strands [6]. Tumor progression continued, and the patient was started on temozolomide. According to Zhang et al., temozolomide is a DNA alkylating agent that induces cell cycle arrest at the G2/M phase, causing apoptosis [7]. The patient’s tumor continued to progress, and she was subsequently started on bevacizumab. 

Unlike the previous treatment, bevacizumab is immunotherapy aiming to prevent pathologic angiogenesis. According to Wilson, pathologic angiogenesis enhances the growth of solid tumors and causes chronic inflammation, cartilage damage, atherosclerotic plaque formation, and scarring of the eye [8]. Wilson also postulates that abnormal angiogenesis starts with an angiogenic switch, a poorly understood mechanism, that upregulates the production of proangiogenic proteins such as vascular endothelial growth factor (VEGF), basic fibroblast growth factor, interleukin-8, platelet-derived endothelial growth factor; and downregulates antiangiogenic proteins such as thrombospondin, endostatin, and angiostatin [8]. Bevacizumab is a monoclonal antibody that binds circulation VEGF, hence preventing the binding of VEGF to its cell surface receptor and curtailing pathologic angiogenesis. Cancer angiogenesis research has shown that early angiogenesis therapy is more successful than late treatment. This is explained mainly by the fact that tumor blood vessels produce more varied growth factors later in disease than they do earlier, meaning patients need drugs against multiple growth factors. However, it is known that antiangiogenic drugs typically only target just one or two growth factors. Combining angiogenic therapy with other interventions such as chemotherapy, radiation therapy, or vaccine therapy may be most effective overall. 

Tumor progression continued despite bevacizumab, and the patient was started on gemcitabine and docetaxel. As reported by de Sousa Cavalcante et al., the most common mechanism of action of gemcitabine is by inhibiting DNA synthesis [9]. Per Farha et al., docetaxel works by both inhibiting microtubular depolymerization and attenuating the effects of BCL-2 and BCL-XL gene expression [10]. Tumor progression continued despite this treatment, at which point, the patient requested to take a break. Per Marwah et al., surgery and chemotherapy can give longer survival even in recurrent disease [11]. Therefore, patients who opt out of treatment may be monitored and provided palliative care. Nishida et al. reported that the five-year survival rate for stage I immature teratoma is 90% to 95%, whereas this survival rate drops to 50% for grade 1 to 2 cancers and to 25% or less for grade 3 tumors [1]. 

## Conclusions

Immature teratomas mainly affect young female patients. Prognosis worsens with delay in diagnosis of disease at later stages. The fertility-sparing approach is the standard of care, and various treatment options should be thoroughly discussed with patients. These options commonly involve surgery and chemotherapy. Immunotherapy with drugs such as Bevacizumab is promising and most effective when initiated early. The five-year survival rate is excellent for stage I immature teratomas, but this survival rate decreases drastically for grade 1, 2, and 3 tumors.
